# Book and Pamphlet Notices

**Published:** 1858-08

**Authors:** 


### BOOK AND PAMPHLET NOTICES.

Transactions of the Twenty-ninth Annual Meeting of the Tennessee State Medical Society, held at Nashville, April 6, 1858.

It would seem, from the very few present at the meeting, that the great importance of such associations is not appreciated by the physicians of Tennessee; this is the case elsewhere, as we have reason to know, and is very much to be regretted. Nothing has contributed to elevate the standard of medical decorum so much within the last few years as the frequent association of physicians. They know each other better, understand what is expected of each individual to the other, and are stimulated by the spirit of laudable ambition thus begotten, to make greater and nobler efforts in behalf of the *common cause.* Before the organization of the American Medical Association or our State and County Medical Societies, comparatively very little of a definite character was known by the mass of the profession about etiquette, ethics, good manners, professionally, etc., etc. Frequent conferences, however, of late have elicited the effect of different practices upon the standing and harmony of the profession, and established an enlightened and liberal code of ethics under which we may all live and associate in amity. If this was all that had been accomplished by these modern institutions, it would have been enough in all conscience to make us feel and take a deep interest in the sustenance of them and their influence; but this is not near half their benefits. Why, how many men in the ranks who could be seen only by close scrutiny along the line, after his name had been privately suggested by a friend, that now stand forth majors, and their names honorably and familiarly known from one side of our broad continent to the other, that must ever have remained destitute of even favorable mention but for the stimulus, fostering aid and publicity given them by professional societies? Writers of no mean character have been *developed* in this way. And then papers that have increased the reputation of the profession abroad would not have had their existence but for these societies. Trains of thought and research have been opened up and discoveries made that will live forever, that else would have lain dormant, perhaps, until the end of time. And the teachings of these associations are of incalculable benefit, particularly the smaller ones; the discussions and facts elicited always benefit some member; and we think it would not be extravagant to say, that every two hours' conference of twenty earnest and devoted medical men on medical subjects will always save twenty lives, by increasing the capacity of all for the efficient discharge of their duty toward the sick. Is there any objection to them? If there is one really valid reason why they should not be sustained, we cannot summon it before our minds this morning. Nothing but indolence prevents the large majority of physicians from attending. All sensible, reflecting men will attribute habitual negligence of these institutions on the part of any member of the profession to downright, unpardonable laziness. It can be nothing else. And it will never fail to demonstrate itself, that such negligence, such laziness, will eventually ruin the delinquent. But to return to the Transactions of the Tennessee State Medical Society; the discussion, as recorded in the minutes, show both spirit and talent in the few members present.

There are only five papers contained in the Transactions, all very short, but practical and well written. The first is by J. L. Maddin, M. D., of Nashville, on the question, How does chloroform cause death? He states that there have been about one hundred cases of death from chloroform altogether; that these, without exception, occurred in operation in minor surgery; no fatal cases in obstetrics. The main practical point in the paper is, that the *local* anesthetic effect of the agent on the nerves of the lungs destroys the *besoian de respeirer*, and thus prevents the reflex stimulus upon the muscles of respiration. That instead of its control being exerted as a destructive agent more powerfully on the nervous centres directly, he thinks the respiratory act ceases from a complete annul of the organic sensitiveness of the lungs. This is, at least, very plausible, and may be true. As a remedy, he recommends artificial respiration until the chloroform gas is all evolved from the respiratory cavity and the anesthetic effect wears off.

The second paper is from Dr. Eve, giving an account of a patient who swallowed a set of artificial teeth. It has traveled the round of the medical journals so generally, that it is not necessary to notice it at any length. It is a curiosity.

The third paper is by L. M. Woodson, M.D., Sumner Co., Tenn., on a case of disease of Cerebro-Spinal Centres, characterized by constant hiccough, followed by death. It loses most of its interest from the fact that a post-mortem could not be procured.

The fourth paper is by W. P. Moore, M.D., of Linwood, Md., on Obstetric Medicine. The author relates several interesting but not extraordinary cases of obstetrics, and winds up with some remarks upon the different operative modes of terminating difficult parturition.

The fifth paper is by C. K. Winston, M.D., of Nashville, and is made up of three cases of Traumatic Tetanus, successfully treated by large doses of sul. morphia and inunction of sweet oil. The morphia was given in quantities sufficient to control the spasms, which required it to be increased each successive day. Three recoveries of this dreadful disease from large and increasing doses of morphia ought to be remembered.

We hope next year to be able to greet a larger volume of as good material from the Tennessee State Medical Society, and see in their record an evidence of an increasing interest on the part of the profession at large in regard to an organization calculated to do so much good. W. H. B.

We have received the first number of the *Medical Journal of North Carolina*, August, 1858, edited by Edward Warren, M.D., and published under the auspices of the State Medical Society, at Edenton, N. C. It is a neat, well arranged and comely periodical, and under auspices of the whole profession of the State; and considering the fact that there is no other in the State, and that its editor is widely and favorably known to the medical world, we think and hope it will have an abundant success. We willingly comply with the request to "exchange."

W. H. B.

TILDEN & CO.'S BOOK OF FORMULA.—This is intended as a guide in the use of their preparations, and the compounding the officinal tinctures and solutions from their fluid and solid extracts. It will afford much information to persons desiring it in this connection. We have also received several numbers of his Journal of Materia Medica, which may be had by sending your name to them at New Lebanon, N. Y. With these were several specimens of sugar-coated pills and granules. We have already drawn the attention of our readers to these valuable preparations as supplying a desideratum in such times of fastidious taste as these are; a trial of them will pay. W. H. B.

